# Chronic Cholecystitis: Anatomical Variants, Pediculitis, and a Candidate Preoperative Framework for Difficult Laparoscopic Cholecystectomy

**DOI:** 10.3390/diagnostics16081201

**Published:** 2026-04-17

**Authors:** Georgiana-Andreea Marinescu, Sarmis Marian Sandulescu, Dumitru Radulescu, Oana Taisescu, Emil-Tiberius Trasca, Elena-Irina Caluianu, Dorin Mercut, Razvan Mercut, Eleonora Daniela Ciupeanu-Calugaru, Alexandru Stefarta, Patricia-Mihaela Radulescu, Citto Taisescu

**Affiliations:** 1 Doctoral School, University of Medicine and Pharmacy of Craiova, 2 Petru Rareș Street, 200349 Craiova, Romania; marinescu_georgiana.andreea@yahoo.com; 2 Department of Surgery, University of Medicine and Pharmacy of Craiova, 2–4 Petru Rares Street, 200349 Craiova, Romania; sarmis.sandulescu@umfcv.ro (S.M.S.); dr_radulescu_dumitru@yahoo.com (D.R.); rui_costa23@yahoo.com (E.-I.C.); mercutdorin@yahoo.com (D.M.); 3 Department of Human Anatomy, University of Medicine and Pharmacy of Craiova, 2–4 Petru Rares Street, 200349 Craiova, Romania; 4 Department of Plastic and Reconstructive Surgery, University of Medicine and Pharmacy of Craiova, 200349 Craiova, Romania; razvan.mercut@umfcv.ro; 5 Department of Biology and Environmental Engineering, University of Craiova, 200585 Craiova, Romania; ciupeanudaniela@gmail.com; 6 Department of Dental Technology, University of Medicine and Pharmacy of Craiova, 200349 Craiova, Romania; alexandru.stefarta@umfcv.ro; 7 Department of Pneumology, University of Pharmacy and Medicine Craiova, 200349 Craiova, Romania; patricia.radulescu@umfcv.ro; 8 Department of Internal Medicine, University of Medicine and Pharmacy of Craiova, 2 Petru Rares Street, 200690 Craiova, Romania; citto.taisescu@umfcv.ro

**Keywords:** laparoscopic cholecystectomy, chronic cholecystitis, biliary anatomical variation, cystic pediculitis, frozen Calot’s triangle, biliary colic, conversion, intraoperative complications, inflammatory biomarkers, conceptual framework

## Abstract

Preoperative risk stratification for laparoscopic cholecystectomy (LC) remains imperfect, particularly in patients with chronic inflammatory remodeling and biliary anatomic variants. Existing tools often focus on acute presentations or intraoperative variables, resulting in uncertainty on how congenital anatomy, recurrent biliary colic, and cystic pediculitis interact. We synthesize a hypothesis-generating conceptual framework and propose an illustrative candidate preoperative rubric for future validation. We performed a structured narrative review of PubMed, Scopus, and Web of Science (January 1990–December 2024; last search: 15 December 2024). Eligible primary studies evaluated clinical history, imaging-defined anatomy, inflammatory biomarkers, and/or operative outcomes (conversion, intraoperative complications, or operative difficulty) in the setting of LC. Acute cholecystitis and chronic/elective cohorts were interpreted separately during the narrative synthesis. Two reviewers screened titles/abstracts and assessed full texts using predefined inclusion/exclusion criteria; due to heterogeneity, no meta-analysis and no formal risk-of-bias tool were applied. The literature supports a plausible vicious cycle in which biliary anatomic variants may impair drainage and promote stasis, recurrent biliary colic, and chronic inflammation, ultimately leading to fibrosis/pediculitis and a “frozen” Calot’s triangle. We translate these signals into an illustrative candidate rubric (0–16 points) spanning three domains: clinical history (0–6), imaging (0–6), and inflammatory biomarkers (0–4). Weights and cut-offs (low: 0–4; moderate: 5–9; high: 10–16) were chosen a priori for conceptual clarity and are not data-derived. This review provides a conceptual map and a candidate variable set to support hypothesis generation, standardized data collection, and staged validation. The rubric is not validated and must not be used for clinical decision-making. Planned next steps include feasibility-oriented derivation, followed by prospective multicenter external validation and impact assessment.

## 1. Introduction

Most published predictors of operative difficulty emphasize classical clinical factors such as older age, comorbidity burden, obesity, and recent or severe acute cholecystitis, and are often derived from acute-care or short-stay/day-surgery workflows [1,2,3]. In contemporary large series, conversion to an open approach is required in a minority of cases (often reported around 3–5%), commonly due to poor visualization, dense adhesions, bleeding, or concern for bile duct injury [4,5]. However, a substantial subset of patients present with long-standing, recurrent biliary colic and chronic remodeling without a typical “acute” phenotype, and the evidence relevant to these patients is dispersed across clinical series, imaging-anatomy studies, and biomarker studies [6,7]. Accordingly, evidence derived from acute cholecystitis cohorts should not be interpreted as directly interchangeable with evidence from chronic or elective disease cohorts because the inflammatory substrate, timing of surgery, and intraoperative decision-making may differ substantially.

Congenital anatomic variants of the gallbladder and extrahepatic biliary tract (e.g., cystic duct course and insertion variants, accessory ducts, septations, duplication) may impair bile flow, promote biliary stasis, and facilitate early stone formation [8,9]. Recurrent biliary colic may then act as a repeated inflammatory trigger, potentially establishing a self-reinforcing cascade toward chronic cholecystitis [10,11,12].

A key downstream manifestation of this chronic process is cystic pediculitis—persistent inflammation and fibrosis around the cystic duct and artery within Calot’s triangle. When advanced, this may result in a “frozen Calot’s triangle,” where tissue planes are obscured and safe identification of biliary structures becomes challenging, thereby increasing the risk of major complications such as bile duct or vascular injury [9,11].

In the context of laparoscopic cholecystectomy, a “difficult gallbladder” may be understood as a clinical–anatomical condition in which safe dissection is hindered by inflammation, fibrosis, adhesions, distorted biliary landmarks, impacted stones, or congenital anatomical variants, thereby threatening successful completion of the critical view of safety technique and increasing the likelihood of subtotal cholecystectomy, conversion, prolonged operative times, or bile duct/vascular injury.

In parallel, systemic inflammatory indices derived from routine blood tests (e.g., NLR, PLR, SII/SIRI) and C-reactive protein have been associated with inflammatory severity and operative difficulty in some cohorts, but their optimal thresholds and incremental predictive value remain uncertain across heterogeneous study designs [13,14,15].

This article is intentionally framed as a structured narrative review and hypothesis-generating conceptual framework. Rather than presenting a clinically validated scoring system, we (i) articulate a ‘vicious cycle’ model linking anatomy, stasis, recurrent pain, chronic inflammation, and pediculitis; (ii) propose an illustrative candidate preoperative rubric to standardize variable collection in future studies; and (iii) outline a staged validation roadmap that must precede any clinical adoption.

## 2. Materials and Methods

We conducted a structured narrative review to identify and synthesize evidence linking chronic cholecystitis phenotypes (including recurrent biliary colic), biliary anatomic variants, systemic inflammatory markers, and operative outcomes during laparoscopic cholecystectomy. PubMed, Scopus, and Web of Science were searched for articles published between January 1990 and December 2024 (last search: 15 December 2024). Search terms combined concepts of chronic cholecystitis, biliary anatomy/variants, pediculitis or fibrosis, inflammatory indices (e.g., NLR, PLR, SII/SIRI), conversion, operative difficulty, and complications. Reference lists of key papers were also screened to identify additional relevant primary studies.

### 2.1. Study Selection

Studies were included based on the following criteria:

• Peer-reviewed articles investigating the association between congenital anatomical anomalies of the gallbladder, recurrent biliary colic, and the development of pediculitis in the context of laparoscopic cholecystectomy.
• Research assessing intraoperative outcomes—such as conversion rates to open surgery and the incidence of operative complications—using imaging modalities, clinical evaluations, and histopathological analyses.

Exclusion criteria were:

• Editorials, letters to the editor, conference abstracts, and reviews lacking original data.
• Studies with incomplete data regarding imaging, clinical, or biological assessments.

Because the included literature was highly heterogeneous with respect to study design, patient phenotype, predictor class, and outcome definition, no formal risk-of-bias scoring instrument was applied. Accordingly, the present synthesis should be interpreted as a structured narrative and hypothesis-generating review rather than as a graded comparative assessment of study quality.

Following title/abstract screening against the predefined criteria, full texts were assessed independently by two reviewers; disagreements were resolved by consensus with a third reviewer. Data items extracted included study design, setting, sample size, preoperative variables (clinical, imaging, anatomical, and biomarker measures), and outcomes related to conversion, operative difficulty, and complications.

To reduce interpretive overlap, studies involving acute cholecystitis and those involving chronic or elective gallbladder disease were considered separately during the narrative synthesis. Acute cholecystitis studies were retained primarily as contextual evidence regarding inflammatory severity, operative difficulty, and bailout strategies, whereas the central conceptual framework of this review focuses on chronic inflammatory remodeling, recurrent biliary colic, and their potential association with difficult laparoscopic cholecystectomy.

Application of these criteria yielded 193 records for full-text review; 30 primary studies met inclusion and were synthesized. Studies excluded after full-text review did not meet the predefined criteria for original data relevance, completeness of imaging/clinical/biological information, or direct relevance to the predefined preoperative variables and operative outcomes of interest. The bibliography is larger because additional sources are cited for background/justification only and are not counted among included primary studies. Additional references introduced during revision to address specific reviewer/editor queries were used only as targeted contextual support and were not incorporated into the original set of 30 primary studies.

**Figure 1:**
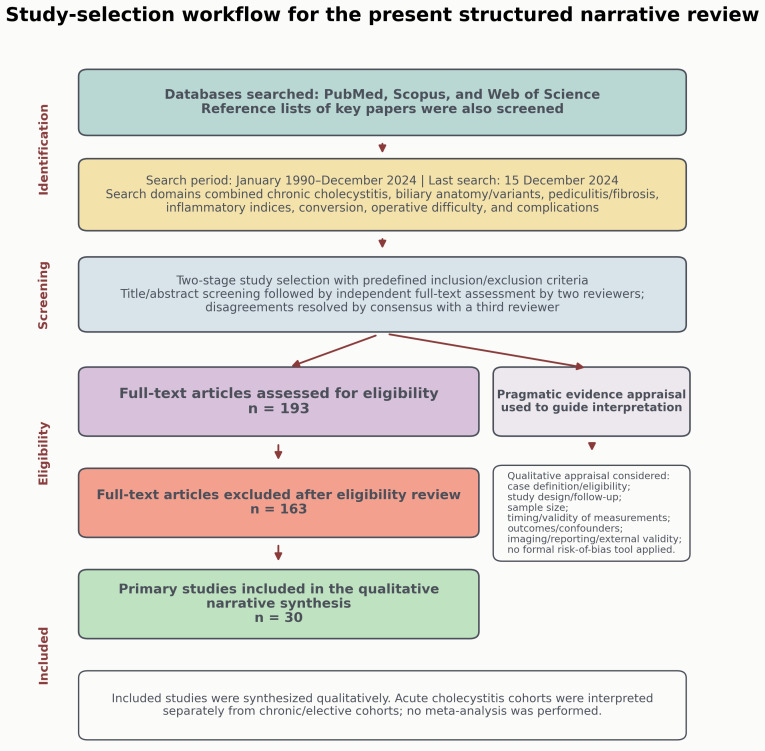
Reference review and study-selection process for the present structured narrative review. The figure summarizes the databases searched, search period, two-stage screening workflow, full-text eligibility review, exclusion after eligibility assessment, inclusion of 30 primary studies, and the separate pragmatic appraisal process used to interpret heterogeneous evidence. Only study-selection counts derived from the original literature search are shown; contextual references added during revision are not included in these counts.

For transparency, the reference review and study-selection process is summarized in Figure 1, and the 30 primary studies included in the structured narrative synthesis are listed in Table 1.

This work was not designed as a systematic review or a scoping review (no protocol registration and no PRISMA-ScR checklist). Given substantial heterogeneity in study designs, populations, variable definitions, endpoints, and clinical phenotype (acute versus chronic presentations), we performed a qualitative narrative synthesis rather than meta-analysis. Particular care was taken not to interpret acute cholecystitis cohorts as directly equivalent to chronic elective gallbladder disease cohorts. To improve transparency, we prespecified eligibility criteria, used dual screening, and report study-selection counts; nevertheless, selection bias and incomplete capture of the literature remain possible.

The search and review process therefore involved database identification, title/abstract screening, independent full-text eligibility assessment, and subsequent qualitative appraisal of the included studies.

### 2.2. Evidence Appraisal and Methodological Considerations

Study appraisal was performed after eligibility selection and was intended to guide interpretation of heterogeneous evidence rather than to exclude studies on the basis of a formal scoring system.

No formal risk-of-bias or study-quality scoring tool (e.g., Newcastle–Ottawa Scale, QUADAS) was applied. Instead, we performed a pragmatic qualitative appraisal to guide interpretation, which considered: (i) clarity of case definition/eligibility; (ii) study design (prospective vs. retrospective) and completeness of follow-up; (iii) sample size; (iv) timing and validity of preoperative measurements (clinical, imaging, biomarkers); (v) outcome definition and relevance (conversion, operative difficulty, complications); (vi) handling of confounders (basic adjustment/stratification when reported); and (vii) reporting quality of imaging protocols (e.g., US/MRCP), risk of population overlap, and external validity.

Importantly, the candidate rubric presented below is an illustrative translation of aggregated literature signals; it has not been derived or validated. All point allocations and thresholds are arbitrary and are included solely for hypothesis generation and validation planning.

### 2.3. Data Synthesis and Development of the Conceptual Framework and Candidate Rubric

Evidence was synthesized qualitatively and organized into a conceptual ‘vicious cycle’ linking anatomic variation, biliary stasis, recurrent biliary colic, chronic inflammation, fibrosis/pediculitis, and operative risk. We then translated recurring literature signals into a candidate preoperative variable set and an illustrative point-based rubric. This rubric is presented for hypothesis generation and study planning only; it is not empirically derived, calibrated, or validated for clinical use.

## 3. A New Etiologic Paradigm: The Role of Congenital Anatomical Anomalies

### 3.1. Distinguishing Classical Metabolic Factors from Anatomical Variations

Historically, the development of gallstones and chronic cholecystitis was attributed primarily to metabolic factors—namely, obesity, rapid weight loss, and dyslipidemia—which were encompassed within the traditional “four F” model [14]. These concepts were fundamental in understanding biliary pathology based on the premise that excess cholesterol or endocrine dysfunction predisposes patients to stone formation. However, as imaging techniques have advanced and clinical studies have evolved, it has become apparent that not all patients with recurrent biliary colics or chronic cholecystitis exhibit an altered metabolic profile. A growing subset of patients—particularly younger, normoponderal individuals—experience repeated biliary pain, suggesting that, in addition to metabolic imbalances, anatomical factors play a crucial role [16,36]. These findings indicate that the predisposition to biliary disease may also be rooted in congenital variations of the gallbladder and biliary system, thereby supporting a hybrid etiologic model in which both metabolic and anatomical factors contribute to pathogenesis.

### 3.2. Types of Anatomical Anomalies of the Gallbladder and Biliary Tract

The structure of the gallbladder and bile ducts varies significantly among individuals, as demonstrated by numerous autopsy and imaging studies. The gallbladder does not always conform to its classic morphological pattern; congenital malformations such as septations—where an internal septum divides the lumen into compartments—or duplication, though rare, can have substantial implications for biliary function [16,17]. These structural variations may lead to inefficient bile evacuation, thereby promoting gallstone formation and recurrent biliary colic [8,18,19].

Of particular importance are variations in the cystic duct. Such anomalies extend beyond mere differences in length or curvature and can include complex configurations. For instance, a tortuous cystic duct that follows an “S” or “W” shaped course can alter bile flow dynamics, creating stasis points that hinder bile movement and favor stone formation [6,7]. Furthermore, structural variability may result in ectopic insertions, whereby the cystic duct drains not into the common hepatic duct as typically expected, but directly into the right hepatic duct or other atypical locations. These unusual insertions, often overlooked in standard diagnostic evaluations, can complicate intraoperative dissection by obscuring the usual anatomical landmarks, thereby increasing the risk of surgical error and injury [16,20].

From a surgical perspective, the most relevant anomalies are those that alter the expected course or insertion of the cystic duct, create uncertainty regarding the junction with the common hepatic duct, or coexist with atypical gallbladder morphology. Such variants are important not only because of their potential effect on bile flow, but also because they may distort operative orientation and increase the risk of misidentification during dissection [8,16,18,19,21].

### 3.3. Biliary Stasis and the Initiation of Early Colic

When the anatomy of the gallbladder or cystic duct is abnormal, bile evacuation is compromised; stagnant bile promotes nucleation of cholesterol crystals and stone growth [22,23]. Consequently, patients with such anomalies—including variant cystic ducts or septate gallbladder—may present with biliary colic at a younger age, sometimes before classical metabolic risk factors are evident [8,23].

Each episode of biliary colic not only causes significant pain but also triggers a local inflammatory response. The recurrence of these episodes sustains the inflammatory process, resulting in thickening of the gallbladder wall and the development of adhesions, particularly within Calot’s triangle. This interplay between biliary stasis and recurrent inflammation establishes a vicious cycle, wherein colic episodes further intensify inflammation, and persistent inflammation, which, in turn, increases the propensity for subsequent colic events [11,12].

In summary, this new etiologic paradigm underscores the importance of a detailed evaluation of congenital gallbladder and biliary tract variations alongside classical metabolic factors in determining the predisposition to biliary disorders. Structural variability directly impacts bile evacuation and can predispose individuals to recurrent colics, which, through repeated inflammatory episodes, may culminate in complicated chronic cholecystitis. In this context, a more precise preoperative assessment does not imply indiscriminate use of advanced imaging in all patients, but rather a selective combination of standard ultrasonography, second-line anatomical clarification with MRCP when biliary anatomy is uncertain or variant anatomy is suspected, and inflammatory marker assessment to better phenotype patients at risk of chronic inflammatory distortion, difficult dissection, bailout procedures, or conversion. This integrated approach has the potential not only to reduce intraoperative complications but also to optimize the overall management of biliary disease, thereby paving the way for a more personalized approach to biliary surgery [17].

## 4. Vicious Cycle: Recurrent Abdominal Colics and Chronic Inflammation

### 4.1. Inflammatory Mechanisms Triggered by Recurrent Biliary Colics

Recurrent biliary colics are not merely painful episodes but initiate an acute inflammatory cascade—characterized by mucosal edema and microvascular injury—that progresses into chronic inflammation through collagen deposition and fibrin organization, as graded in operative series [9]. Moreover, systemic inflammatory indices (e.g., NLR, PLR, SII) have been associated with inflammatory severity and operative difficulty, particularly in acute cholecystitis cohorts; in the present review, these data are interpreted as contextual rather than directly equivalent evidence for chronic remodeling pathways [14,15,21]. This remodeling process stiffens the gallbladder wall and creates an environment conducive to further bile stasis, thereby perpetuating the cycle in which each episode of colic intensifies inflammation, which in turn predisposes to additional colic events [10,12].

### 4.2. Inflammatory Markers and Their Role in Assessing Chronic Inflammation

The objective assessment of systemic inflammation is achieved through measurement of specific inflammatory markers that provide critical insights into the inflammatory response. The neutrophil-to-lymphocyte ratio (NLR) is a widely used inflammatory indicator; elevated values have been associated with more severe inflammatory burden, particularly in acute calculous cholecystitis cohorts. In the context of this review, such markers are discussed as potentially informative adjuncts for chronic inflammatory remodeling, but not as validated discriminators between acute and chronic disease states. Similarly, the platelet-to-lymphocyte ratio (PLR) and C-reactive protein (CRP) levels may contribute to evaluating inflammatory burden, although their interpretation may differ between acute and chronic clinical settings. Even in the absence of overt clinical symptoms, elevated CRP reflects a continuously active inflammatory process, and when combined with a history of recurrent biliary colics, these markers may help identify patients at risk for severe complications, including the development of complicated chronic cholecystitis [14,15].

### 4.3. Transformation of the Gallbladder: From Acute Colics to Complicated Chronic Cholecystitis

Recurrent biliary colics may contribute to a progressive transformation of the gallbladder through repeated inflammatory insults. Although each episode may include an acute inflammatory component, the focus of the present framework is the cumulative remodeling that characterizes chronic disease rather than the clinical entity of acute cholecystitis itself. Over time, ongoing inflammation may lead to morphological changes such as gallbladder wall thickening and progressive fibrosis, which can contribute to a difficult dissection and a “frozen Calot” scenario in advanced cases [10,11,36]. Severely contracted/sclerotic gallbladder phenotypes have also been discussed as potentially higher-risk lesions in relation to gallbladder carcinogenesis, although the absolute risk is variable across populations and study designs [23,36].

This evolution reflects not only the cumulative effect of repeated inflammation but also the pathological adaptation of the tissue to mechanical and biochemical stresses induced by recurrent colics. The resultant remodeling diminishes the gallbladder’s elasticity and may impair its contractile function, thereby hindering efficient bile evacuation. Consequently, complicated chronic cholecystitis not only results in significant patient discomfort but also poses a major surgical challenge as distorted anatomy and inflammatory scarring can markedly increase the difficulty of dissection and the likelihood of conversion or bailout strategies [6,10].

### 4.4. Clinical Implications and Surgical Challenges

Clinically, the impact of this vicious cycle of recurrent biliary colics and chronic inflammation is profound. Patients presenting for surgery under these conditions often exhibit advanced cholecystitis, characterized by dense adhesions and marked fibrosis. These pathological changes cause significant preoperative discomfort and present formidable challenges intraoperatively. During laparoscopic cholecystectomy, the presence of a “frozen Calot’s” complicates the identification of critical anatomical structures—such as the cystic duct and common hepatic duct—substantially increasing the risk of iatrogenic injuries, including inadvertent damage to bile ducts or blood vessels [1,5,28,37].

**Figure 2:**
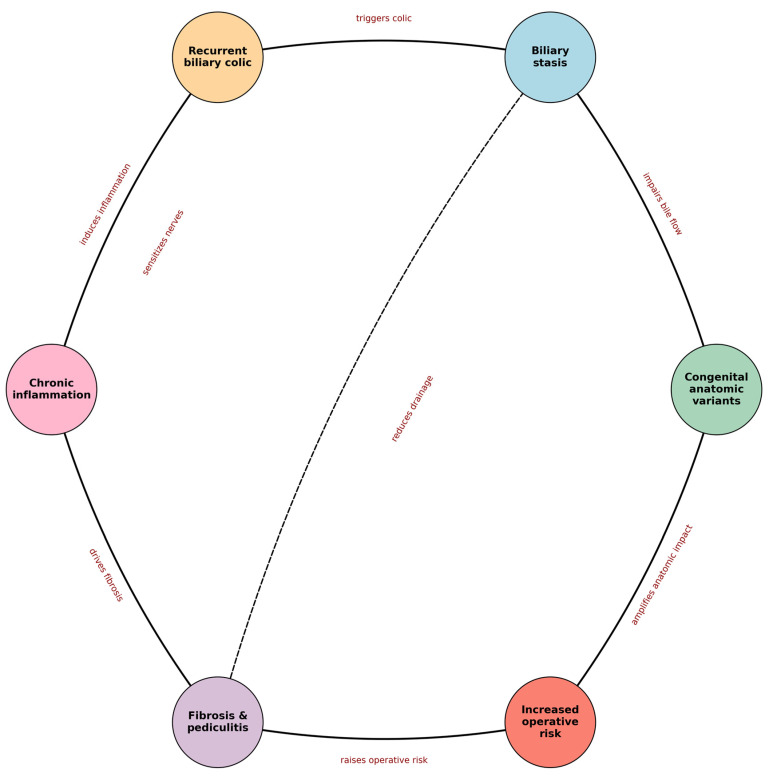
Conceptual framework of the proposed ‘vicious cycle’ in chronic cholecystitis (hypothesis-generating). Solid arrows indicate the main proposed pathway; dashed arrows indicate reinforcing feedback loops.

For surgeons, recurrent biliary colic may progressively remodel the gallbladder and Calot’s triangle, setting the stage for a more complex operation. Figure 2 summarizes the hypothesis-generating ‘vicious cycle’ framework that motivates the candidate preoperative variables proposed in Section 7.

Furthermore, meticulous preoperative planning—incorporating the evaluation of inflammatory markers and high-resolution imaging—is vital for optimizing surgical safety and reducing the risk of iatrogenic injury.

## 5. Pediculitis and Alterations in Calot’s Triangle

### 5.1. Definition and Significance of Cystic Pediculitis

In the present manuscript, “cystic pediculitis” is used as a descriptive term referring to chronic inflammation localized to the cystic pedicle—a region comprising the cystic duct, cystic artery, and the adjacent connective tissue within Calot’s triangle [8,11,18]. Over time, recurrent biliary colic episodes and sustained inflammation transform normally elastic tissue into a rigid, fibrotic structure. Essentially, cystic pediculitis marks the juncture at which the inflammatory response becomes persistent and reparative mechanisms are excessively activated, leading to collagen deposition and fibrin organization. The end result is the formation of an inflammatory “nodule” that fails to regenerate properly, thereby complicating subsequent surgical dissection [8,11,18].

### 5.2. The “Frozen Calot” Phenomenon

A direct consequence of cystic pediculitis is the “frozen Calot” phenomenon. In this scenario, chronic inflammation and marked fibrosis lead to extensive adhesions within Calot’s triangle, resulting in an almost complete loss of recognizable anatomical landmarks. The cystic duct—and, at times, the common bile duct—may become densely adherent to surrounding structures, creating a fused tissue mass. For the surgeon, this significantly complicates dissection and increases the risk of iatrogenic injuries (e.g., bile duct injury or hemorrhage). Because this situation is often not anticipated preoperatively, intraoperative bailout strategies (e.g., subtotal cholecystectomy, fundus-first dissection, enhanced biliary imaging) may be required to operate safely [5,7,25,26].

### 5.3. Xanthogranulomatous Cholecystitis and the Spectrum of Chronic Inflammation

In cases of severe chronic inflammation, some patients may progress to more complex entities such as xanthogranulomatous cholecystitis (XGC). XGC is characterized by extensive inflammatory infiltration and fibrosis, frequently causing dense adhesions and marked thickening of the gallbladder wall, and may mimic malignancy. In such circumstances, dissection within Calot’s triangle can be extremely difficult, and conversion to open surgery is reported more frequently than in standard LC. For example, in the cited 2021 series, laparoscopic cholecystectomy was initially attempted in 69 patients with XGC, and conversion to open cholecystectomy occurred in 26.09% of cases; intense fibrosis accounted for 66.7% of conversions, and increased gallbladder wall thickness together with acute cholecystitis were identified as statistically significant risk factors (*p* < 0.05) [27]. Accordingly, XGC represents the extreme end of the chronic-inflammation spectrum in which recurrent inflammatory injury can culminate in profound anatomic distortion and elevated operative risk [11,18].

## 6. Impact on Surgical Interventions

### 6.1. Conversion Rates and Intraoperative Complications

The success of a laparoscopic cholecystectomy largely depends on the clear identification and accessibility of biliary structures. Unfortunately, in patients with chronic inflammation and a “frozen Calot,” accurately identifying the cystic duct and the common hepatic duct becomes extremely challenging. Consequently, the risk of converting to open surgery escalates, and intraoperative complications frequently manifest as inadvertent injuries to the bile ducts or blood vessels [5]. In such circumstances, difficulties in dissection may lead to uncontrollable bleeding or accidental transection of the common bile duct—events that demand immediate surgical intervention to ensure patient safety [6,37]. Studies have shown that, in broad contemporary laparoscopic cholecystectomy series, reports of conversion to open surgery are generally in the low single-digit range, commonly around 3–5% [3,4,6,24,28]. In the Swiss prospective 3-year study cited in our bibliography, the cohort included 10,174 patients, underscoring the scale of evidence supporting this baseline estimate. However, the risk increases substantially when severe chronic inflammation, dense adhesions, “frozen Calot,” or distorted biliary anatomy impair safe identification of the cystic structures [24]. In more advanced chronic inflammatory phenotypes, the magnitude of this problem becomes clearer. For example, in the cited 2021 series on xanthogranulomatous cholecystitis, 69 patients underwent an initial laparoscopic approach, and conversion to open cholecystectomy occurred in 26.09% of cases; the most common reason was intense fibrosis (66.7%), while increased gallbladder wall thickness and acute cholecystitis were statistically significant risk factors on ultrasonography (*p* < 0.05) [27]. These data better illustrate that conversion risk is not merely “increased” in difficult chronic inflammatory disease, but may rise well above standard LC ranges in selected high-risk phenotypes.

### 6.2. Alternative Dissection Approaches and Techniques

Because difficult dissection is often only fully appreciated intraoperatively, a range of “bailout” approaches has been developed to preserve safety when safe dissection cannot be achieved. Rather than forcing dissection in a hostile Calot’s triangle, surgeons may adopt subtotal cholecystectomy (fenestrating or reconstituting variants), which can reduce the risk of major bile duct injury at the cost of a higher likelihood of postoperative biliary events or reintervention in some series [5,25,29].

Additional techniques include fundus-first (top-down) dissection, careful use of energy devices, and early conversion to an open approach when exposure and control remain unsafe [3,4,6]. Importantly, these strategies should be framed as context-dependent options rather than algorithmic rules, and they do not replace the need for validation of preoperative risk stratification frameworks [26,29,30].

Intraoperative imaging adjuncts may also support safer navigation of variant anatomy. Standard intraoperative cholangiography and indocyanine green (ICG) fluorescent cholangiography have been associated with improved biliary structure visualization and surgeon confidence, although reported benefits vary by workflow, timing, and case mix; randomized data exist in elective settings [30,31,38]. In the context of a hypothesis-generating rubric, these techniques are best discussed as potential mitigators of operative risk rather than mandated interventions.

### 6.3. The Role of Surgical Experience and Team

Beyond patient and disease factors, surgeon experience and team familiarity with difficult cholecystectomy workflows are consistently linked to conversion and complication rates. In the retrospective BMC Surgery study already cited in our manuscript, 4013 patients underwent elective laparoscopic cholecystectomy, and conversion to open surgery occurred in 67 cases (1.7%). In that analysis, surgeon experience was identified as an independent predictor of conversion, together with male sex, older age, higher BMI, prior upper abdominal surgery, gallbladder wall thickening, and previous acute cholecystitis. These findings support the view that conversion is influenced not only by patient-level risk factors but also by operator experience and judgment during difficult dissection [3].

For high-complexity cases, a structured team approach—preoperative briefing, availability of appropriate imaging adjuncts, and readiness for conversion or subtotal techniques—may improve safety. This interpretation is also consistent with pooled safety analyses showing that major bile duct injury after laparoscopic cholecystectomy has remained a persistent concern over time, generally reported in the range of approximately 0.32–0.52%, thereby reinforcing the importance of training, institutional pathways, and safe-dissection culture in difficult cases [7,30].

Accordingly, the proposed rubric is intended to support anticipation and planning (e.g., case scheduling, senior supervision, resource allocation) in future validation studies, rather than to prescribe clinical actions in the current manuscript.

### 6.4. Implications and Conclusions

The combined impact of anatomical and inflammatory factors complicates the execution of a standard laparoscopic cholecystectomy. Severe chronic inflammation and anatomical anomalies are associated with conversion rates that exceed the usual 3–5% reported in standard laparoscopic cholecystectomy series, particularly when dense fibrosis, hostile adhesions, or distorted biliary anatomy compromise safe dissection [3,4,6,24,28]. In selected high-risk chronic inflammatory phenotypes such as xanthogranulomatous cholecystitis, reported conversion rates may reach 26.09% after an initial laparoscopic approach [27]. To manage these challenges effectively, alternative surgical approaches—such as laparoscopic subtotal cholecystectomy, the fundus-first technique, and the use of intraoperative cholangiography—are indispensable.

Furthermore, the experience of the surgical team is paramount; seasoned teams can quickly adapt their operative strategy to the encountered situation, thereby reducing the risk of iatrogenic injuries and major complications. Integrating alternative surgical techniques and advanced technological resources has led to a significant improvement in clinical outcomes and a reduction in postoperative morbidity [5,7,25,26,29,30,31,38].

In conclusion, the presence of chronic inflammation and anatomical anomalies markedly complicates surgical intervention, increasing the difficulty of dissection and the risk of complications. Adopting alternative surgical approaches, alongside the use of intraoperative imaging techniques and leveraging extensive surgical experience, is essential for optimizing operative safety and reducing conversion rates to open surgery. These measures not only improve long-term outcomes but also reduce patient risk, transforming intraoperative challenges into opportunities for safer, more effective surgery [6,24,26,30,37].

## 7. Candidate Preoperative Risk Criteria: An Anatomic–Inflammatory Conceptual Framework

### 7.1. Integration of Clinical Factors

In the context of chronic cholecystitis, traditional risk factors such as obesity and metabolic disturbances do not always fully account for the complexity of challenging surgical cases. Equally important is the patient’s clinical history. For instance, the frequency and severity of biliary colics are direct indicators of repetitive inflammatory processes, which in turn predispose to chronic inflammation and fibrosis. Patients experiencing frequent biliary pain may represent a higher-risk chronic phenotype, particularly when recurrent symptoms are associated with progressive imaging abnormalities or prior inflammatory events. A documented history of acute cholecystitis may be considered a contextual aggravating factor, but acute and chronic pathways should not be interpreted as fully interchangeable clinical entities. When evaluated collectively, these clinical factors provide a clearer picture of a patient’s predisposition to a “difficult gallbladder” [3,6,7].

### 7.2. Imaging Assessment

Modern imaging modalities have revolutionized the evaluation of biliary anatomy. Abdominal ultrasound remains the first-line investigation and is commonly used to assess gallbladder wall thickening, which may suggest chronic inflammatory remodeling. Advanced imaging techniques such as MRCP allow for detailed assessment of biliary and cystic duct anatomical variations (e.g., tortuosity, ectopic insertion, accessory ducts) that can complicate dissection and increase the risk of complications during laparoscopic cholecystectomy [17,18,19,32]. When available, standardized imaging descriptors can be incorporated into a preoperative risk framework to support hypothesis generation and anticipated operative difficulty.

Beyond simple detection of gallstones, ultrasonography may also provide indirect clues to operative difficulty. In the chronic setting, particular attention should be paid to marked wall thickening, a contracted or scleroatrophic gallbladder, reduced distensibility, impacted stones, and distortion of the infundibular/Hartmann’s pouch region, as these findings may suggest advanced inflammatory remodeling, fibrosis, and potentially difficult dissection. Although ultrasound cannot define all anatomical relationships with the precision of cross-sectional cholangiographic techniques, it remains the most practical first-line modality for identifying patients in whom more complex local anatomy should be anticipated.

When biliary anatomy is unclear or a congenital/anatomical variant is suspected, MRCP can provide more detailed delineation of the cystic duct and extrahepatic biliary tree. Variants such as tortuous cystic ducts, low or ectopic insertion, medial insertion, parallel course, accessory ducts, or other atypical configurations may impair operative orientation and increase the risk of misidentification during laparoscopic cholecystectomy [17,18,19,32]. In this context, imaging should not be viewed as a universal escalation step for all patients, but rather as a selective tool for anatomical clarification and preoperative phenotyping in cases considered at higher operative risk.

Hepatobiliary scintigraphy (HIDA scan) may provide complementary functional information in selected cases, particularly when ultrasonography is negative or equivocal despite persistent biliary symptoms, or when cystic duct obstruction or chronic gallbladder dysfunction remains uncertain [39].

### 7.3. Inflammatory Markers and Their Evaluation

Beyond clinical and imaging factors, blood-based inflammatory markers provide additional information regarding the activity of inflammation. The neutrophil-to-lymphocyte ratio (NLR) is a widely studied inflammatory indicator; higher values (commonly >3–4 in published cohorts) have been associated with more severe acute calculous cholecystitis and adverse perioperative outcomes [14,15,21]. In the present manuscript, these studies are interpreted as indirect or contextual evidence, and not as direct validation of biomarker thresholds for chronic elective gallbladder disease. Additional indices—including the platelet-to-lymphocyte ratio (PLR) and composite systemic inflammatory indices (e.g., SII, SIRI)—may further reflect inflammatory burden and are reasonable exploratory candidates for future validation studies [13,36]. C-reactive protein (CRP) and related biomarkers can complement this evaluation as markers of systemic inflammatory activity; however, their optimal preoperative thresholds for predicting operative difficulty or complications require dedicated derivation and validation in well-designed cohorts. Accordingly, these markers are discussed as candidates within the conceptual framework rather than validated decision thresholds.

### 7.4. Conceptual Framework: Illustrative Candidate Rubric (Not Validated)

To translate the narrative synthesis into testable hypotheses, we propose an illustrative candidate rubric that organizes variables across three preoperative domains: clinical history, imaging-defined anatomy/inflammation, and systemic inflammatory markers. The point assignments and risk strata are intentionally illustrative (chosen a priori), and they should be viewed as a template for standardized data capture and subsequent model derivation—not as a ready-to-use clinical score (Table 2).

**Figure 3:**
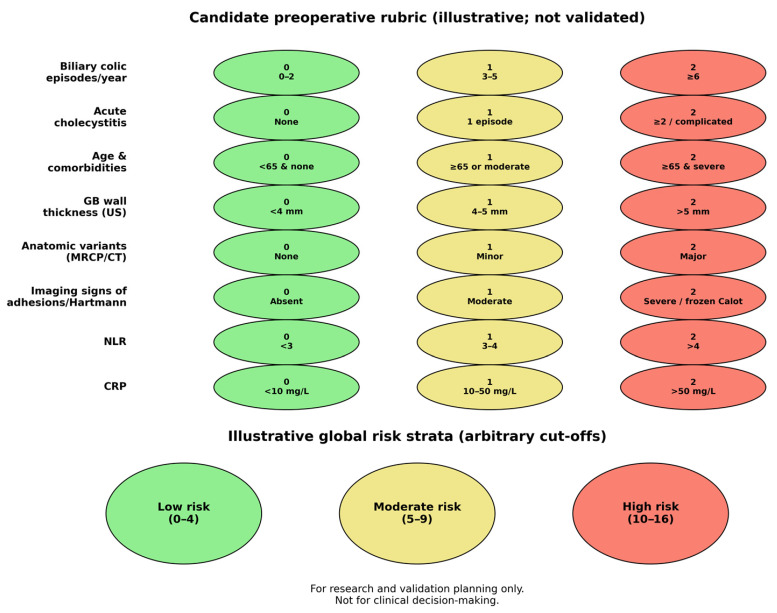
Candidate preoperative rubric elements and illustrative global risk strata (arbitrary cut-offs; not validated).

Illustrative global strata (low: 0–4; moderate: 5–9; high: 10–16) are shown in Figure 3. These thresholds are arbitrary and are included only to support conceptual discussion and future validation planning.

## 8. Validation Roadmap and Context

### 8.1. Necessity of Multicenter Studies

**Figure 4:**
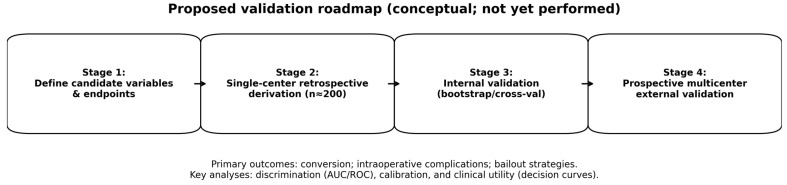
Proposed validation roadmap for staged evaluation of the candidate rubric (conceptual; not yet performed).

For any preoperative model to be clinically meaningful, it must be derived and validated using appropriate study designs and transparent reporting. Accordingly, we propose a staged evaluation plan. First, a single-center retrospective cohort will be used as an initial feasibility-oriented derivation step to pilot variable capture, assess data completeness, and explore preliminary model structure. Because uncommon biliary tract anomalies are unlikely to be adequately represented in a small single-center cohort, definitive derivation and validation will require substantially larger multicenter datasets. Planned analyses include discrimination (ROC/AUC with confidence intervals), calibration (calibration plots and calibration-in-the-large/slope), and internal validation (bootstrap resampling or cross-validation) to estimate optimism. Second, multicenter prospective studies should collect standardized variables (clinical history, imaging descriptors, and biomarkers) using harmonized definitions and should report both discrimination and calibration with prespecified endpoints (conversion, operative difficulty tiers, and intraoperative complications). Only after robust external validation should clinical utility be assessed (e.g., decision-curve analysis and impact studies evaluating changes in workflow, safety endpoints, and resource use). A schematic overview is provided in Figure 4.

The integrated model combines clinical, imaging, and biological factors into a hypothesized preoperative risk framework intended to guide future validation; it is not a validated clinical tool.

### 8.2. Importance of MR Cholangiography and Advanced Imaging

Selective advanced imaging is expected to be informative during validation, particularly in anatomically unclear cases or when biliary variants are suspected. MR cholangiography delineates biliary anatomy, including subtle cystic-duct and bile-duct variants that may affect bile flow; consistent protocols (e.g., sequence parameters and reporting templates) will be important for reproducibility. Intraoperative fluorescence cholangiography can clarify critical structures and, in selected series, has been associated with fewer injuries. Incorporating standardized imaging into the validation plan may improve preoperative risk classification and inform patient-specific operative plans, including selective resource allocation and anticipated bailout strategies [5,18,30].

Compared with conventional cholangiography, MRCP offers the major advantage of non-invasive preoperative mapping of the biliary tree and can depict many cystic-duct and bile-duct variants without cannulation, contrast injection, or ionizing radiation [18,32,40]. By contrast, conventional cholangiography remains valuable when immediate ductal opacification is required intraoperatively or through an existing drainage route because it provides real-time procedural information and may better demonstrate certain fine ductal communications once contrast has directly filled the biliary system [40]. Accordingly, these techniques should be viewed as complementary rather than interchangeable: MRCP is better suited to selective preoperative anatomical delineation, whereas conventional cholangiography is most useful when dynamic intraprocedural clarification is required [40].

In selected high-risk patients with acute cholecystitis, percutaneous cholecystostomy may serve as a temporizing or bridging intervention when immediate cholecystectomy is not feasible. In addition, the cholecystostomy tract may provide access for subsequent cholangiographic assessment in selected cases requiring further biliary evaluation or interval operative planning [41].

### 8.3. Context Relative to Existing Predictive Models

Existing tools for anticipating difficult LC vary in their variable sets and intended settings, and many emphasize acute cholecystitis severity or intraoperative findings. Our framework is deliberately different in that it centers a chronic pathway (recurrent biliary colic → chronic inflammation → pediculitis/frozen Calot) and explicitly incorporates biliary anatomic variants and systemic inflammatory indices as candidate preoperative variables. For this reason, evidence derived from acute cholecystitis prediction models is discussed for context but is not treated as directly interchangeable with evidence relevant to chronic inflammatory remodeling and recurrent biliary colic. Until derivation and validation are performed in representative cohorts, no claims of predictive performance, clinical utility, or superiority over existing approaches can be made.

### 8.4. Clinical Implications and Future Directions

If validated, an integrated preoperative approach could support more consistent operative planning (e.g., anticipating difficult exposure, ensuring appropriate expertise and equipment readiness, and predefining bailout strategies such as subtotal or fundus-first techniques). At present, these implications remain hypothetical. The immediate value of the framework is to organize existing signals, standardize candidate variables, and define a transparent path to validation and potential clinical translation.

Clinical adoption of such a framework may nevertheless face important real-world barriers, including operator dependence of ultrasonography, unequal access to MRCP across institutions, cost and workflow constraints, and variable availability of advanced imaging or hepatobiliary expertise. These implementation factors should be considered in future validation studies and in the broader translation of the proposed framework across centers with different resource levels.

## 9. Limitations of the Study

Several limitations should be emphasized to avoid overinterpretation. First, this work is a structured narrative review rather than a systematic review or scoping review; despite predefined eligibility criteria and dual screening, selection bias and incomplete capture of the literature are possible. Second, the included studies were heterogeneous in design (retrospective vs. prospective), clinical phenotype (acute versus chronic/elective presentations), definitions of operative difficulty, and outcome reporting, which limited direct comparability and required cautious narrative separation of acute and chronic cohorts. Third, no formal risk-of-bias tool or quality scoring framework was applied, limiting the ability to assess the internal validity and relative strength of the included studies and to weight the evidence rigorously across studies. Fourth, the candidate rubric presented here uses a priori weights and arbitrary cut-offs for conceptual clarity; it is not data-derived, calibrated, or validated, and therefore should not be used for clinical decision-making. Finally, publication bias and selective reporting may influence the apparent strength of some associations (particularly biomarker thresholds).

## 10. Conclusions

We propose a hypothesis-generating conceptual framework describing a potential ‘vicious cycle’ in chronic cholecystitis that links biliary anatomic variants, biliary stasis, recurrent biliary colic, chronic inflammation, and cystic pediculitis/frozen Calot’s triangle. To operationalize this framework, we outline a candidate preoperative variable set and an illustrative point-based rubric intended solely for research, standardized data capture, and staged validation. No claims of predictive performance or clinical utility are made, and the rubric must not be used to guide patient care at this stage.

Future work should follow a staged pathway: (i) retrospective single-center derivation with internal validation (discrimination and calibration), (ii) prospective multicenter external validation with harmonized variable and endpoint definitions, and (iii) impact studies assessing whether use of a validated model improves safety, workflow, and resource allocation in laparoscopic cholecystectomy.

## Figures and Tables

**Table 1:** Primary studies included in the structured narrative synthesis (*n* = 30).

Study	Study Type	Main Contribution to the Narrative Synthesis
Ranjan, 2022 [1]	Prospective observational clinical study	LC safety/feasibility in mild–moderate acute cholecystitis; contextual evidence on operative difficulty
Sakpal, 2010 [4]	Clinical outcomes series	Conversion from laparoscopic to open cholecystectomy
Ábrahám, 2021 [3]	Retrospective cohort	Preoperative conversion risk factors and surgeon experience in elective LC
Garg, 2022 [9]	MRCP anatomy study	Cystic duct anatomy and association with cholelithiasis
Chang, 2005 [10]	Operative inflammation grading study	Inflammation severity and operative difficulty
Nesland, 2004 [11]	Pathology study	Chronic cholecystitis ultrastructural/pathologic substrate
Chen, 2024 [13]	Retrospective biomarker study	CBC-derived inflammatory indices in acute versus chronic calculous cholecystitis
Serban, 2023 [14]	Clinical biomarker study	NLR/PLR/SII and severity/adverse outcomes in acute calculous cholecystitis
Lee, 2014 [15]	Retrospective cohort	Preoperative NLR and severe cholecystitis
Hasan, 2013 [16]	Operative anatomy series	Extrahepatic biliary anomalies encountered during cholecystectomy
Chhikara, 2022 [17]	Prospective observational imaging study	Preoperative MRCP versus intraoperative biliary anatomy
Sarawagi, 2016 [18]	MRCP cross-sectional anatomy study	Cystic duct variations and clinical implications
Taghavi, 2022 [19]	Cross-sectional MRCP study	Cystic duct variants on MRCP
Talpur, 2010 [20]	Operative anatomy series	Anatomical variations and congenital anomalies of the extrahepatic biliary system encountered during LC
Prabhu, 2024 [21]	Prospective clinical study	NLR as a marker of severe acute cholecystitis
Murtaza Khomusi, 2022 [22]	Histopathology/clinical series	Histologic diagnoses in patients undergoing cholecystectomy for chronic cholecystitis
Kanoh, 2001 [23]	Pathology/lesion-risk study	Contracted cholecystitis lesions as higher-risk phenotypes
Z’graggen, 1998 [24]	Prospective large cohort	Complications of LC in 10,174 patients
Kaplan, 2014 [25]	Surgical outcomes study	Subtotal versus open total cholecystectomy in complicated cholecystitis
Haverinen, 2025 [26]	Clinical imaging/surgical technique study	ICG fluorescence and difficult fundus-first cholecystectomy
Şimşek, 2021 [27]	Clinical series	Xanthogranulomatous cholecystitis and conversion risk
Domínguez, 2011 [28]	Prospective cohort	Factors associated with conversion during LC
Midya, 2021 [29]	Surgical outcomes series	Re-interventions/readmissions after laparoscopic subtotal cholecystectomy
Symeonidis, 2024 [30]	Randomized controlled trial	Standard IOC versus ICG fluorescent cholangiography
Broderick, 2021 [31]	Clinical outcomes study	Fluorescent cholangiography and LC outcomes
Uddin, 2023 [5]	Clinical study	Bile duct injury occurrence during laparoscopic cholecystectomy
De Filippo, 2008 [32]	MRCP imaging study	Congenital anomalies/variations of bile and pancreatic ducts
Korkmaz, 2024 [33]	Retrospective observational biomarker study	SII and other inflammatory parameters in acute cholecystitis
Kwon, 2004 [34]	Surgical/pathology study	Surgical procedures and histopathologic findings in xanthogranulomatous cholecystitis
Kim, 2013 [35]	Imaging case-based study	Biliary flow impairment in septate gallbladder on hepatobiliary scintigraphy/SPECT-CT

**Table 2:** Candidate preoperative rubric for operative difficulty/conversion risk in laparoscopic cholecystectomy (illustrative; not validated).

Variable	0 Points	1 Point	2 Points
**Clinical history (max 6 points)**			
Biliary colic episodes/year	0–2	3–5	≥6
Prior acute cholecystitis	None	1 episode	≥2 episodes or complicated
Age and comorbidity burden	<65 years and no major comorbidity	≥65 years or moderate comorbidity	≥65 years and severe comorbidity
**Imaging (max 6 points)**			
Gallbladder wall thickness (ultrasound)	<4 mm	4–5 mm	>5 mm
Biliary/anatomic variants (MRCP/CT)	None detected	Minor variant	Major variant/high-risk anatomy
Indirect imaging features of difficult local anatomy (e.g., contracted gallbladder/distorted Hartmann’s pouch region)	Absent	Moderate	Severe/suspected “frozen Calot”
**Inflammatory biomarkers (max 4 points)**			
Neutrophil-to-lymphocyte ratio (NLR)	<3	3–4	>4
C-reactive protein (CRP)	<10 mg/L	10–50 mg/L	>50 mg/L

Note: This rubric is illustrative and hypothesis-generating. Point weights and cut-offs were chosen a priori for conceptual clarity; they are not empirically derived, calibrated, or validated. The rubric should not be used for clinical decision-making.

## Data Availability

No new data were created or analyzed in this study. Data sharing is not applicable to this article.
